# NEK9, a novel effector of IL-6/STAT3, regulates metastasis of gastric cancer by targeting ARHGEF2 phosphorylation

**DOI:** 10.7150/thno.53169

**Published:** 2021-01-01

**Authors:** Guofang Lu, Siyuan Tian, Yi Sun, Jiaqiang Dong, Na Wang, Jiaoxia Zeng, Yongzhan Nie, Kaichun Wu, Ying Han, Bin Feng, Yulong Shang

**Affiliations:** 1 State Key Laboratory of Cancer Biology and National Clinical Research Center for Digestive Diseases, Xijing Hospital of Digestive Diseases, Fourth Military Medical University, Xi'an, China, 710032.; 2 Department of Ultrasound Diagnostics, Tangdu Hospital, Fourth Military Medical University, Xi'an, China, 710032.; 3 Department of Radiation Oncology, Xijing Hospital, Fourth Military Medical University, Xi'an, China, 710032.

**Keywords:** Gastric cancer, Metastasis, NEK9, Inflammation, Phosphorylation

## Abstract

**Rationale:** Inflammatory stimuli from the tumor microenvironment play important roles in cancer progression. However, the mechanism of promotion of cancer metastasis by inflammation in gastric cancer (GC) is poorly understood.

**Methods:** The roles of NEK9 were validated *via* loss-of-function and gain-of-function experiments in vitro and in an animal model of metastasis. Cytoskeletal reorganization-associated molecules were detected by GST pull-down. The regulation of ARHGEF2 by NEK9 was investigated by phosphoproteomics analysis, immunoprecipitation (IP) and in vitro kinase assay. The transcriptional regulation of miR-520f-3p was studied using luciferase reporter and chromatin immunoprecipitation (ChIP). The expression of these proteins in GC tissues was examined by immunohistochemistry.

**Results:** NEK9 directly regulates cell motility and RhoA activation in GC. The phosphorylation of ARHGEF2 by NEK9 is the key step of this process. NEK9 is a direct target of miR-520f-3p, which is transcriptionally suppressed by IL-6-mediated activation of STAT3. A decrease in miR-520f-3p leads to the amplification of IL-6/STAT3 by targeting GP130. A simultaneous elevation of the levels of NEK9, GP130 and p-STAT3 was confirmed in the lymph nodes and distant metastases. An increase in NEK9, GP130 and STAT3 is associated with reduced overall survival of GC patients.

**Conclusion:** This study demonstrates that activation of STAT3 by IL-6 transcriptionally suppresses miR-520f-3p and diminishes the inhibitory effects of miR-520f-3p on NEK9 and GP130. An increase in GP130 enhances this signaling, and NEK9 directly influences cell motility and RhoA activation by targeting the phosphorylation of ARHGEF2. Targeting the IL-6-STAT3-NEK9 pathway may be a new strategy for GC treatment.

## Introduction

Gastric cancer (GC) is the second most common cancer in China and the third leading cause of cancer-related deaths 1, 2. The occurrence of metastasis determines clinical decision making in the treatment of GC, including the feasibility of surgical treatment and targeted therapy. Metastasis is an important factor associated with reduced overall survival in GC patients.

Altered cellular motility is a hallmark feature of metastasis that facilitates the progression of cancer cells to the distant sites 3. Dynamic reorganization of cytoskeleton is the key requirement for the changes in cell motility 4, 5. Reorganization of the cytoskeleton is critical for the morphological changes of the cells involved in active movement 6. The leading edge of the migrating cells contains flat membranous lamellipodial protrusions, where the protrusive force is generated by localized actin polymerization 7. Spatial and temporal regulation of this process is mediated by multiple cellular signaling pathways, and Rho GTPase is a ubiquitous component of the signaling pathways 4, 7, 8. Rho GTPase is associated with the formation of membrane protrusion at the leading edge and the membrane contraction at the rear end, suggesting a fundamental role of this protein in directed cell movement. The functions of Rho GTPase depend on active or inactive states 9-11. The activation of Rho GTPase is regulated by guanine nucleotide exchange factors (GEFs), and inactivation is mediated by GTPase activating proteins (GAPs) 9-11. In addition to the protein level, the activity is an important factor that determines the functions of Rho-GEFs and Rho-GAPs 12-15. The phosphorylation of specific sites by protein kinases can enhance or suppress the activities of Rho-GEFs and Rho-GAPs, leading to distinct regulatory effects on cytoskeletal organization.

Emerging studies have shown that the expression and activity of cytokines are deregulated in many cancer types and contribute to chronic inflammation in tumor microenvironment 16-19. Interleukins, chemokines and lymphokines play distinct roles in cancer cells to sustain aberrant growth and survival 17, 20. Inflammation serves as the driving force for the initiation of cancer metastasis. IL-6 functions by binding to IL-6R subunit, which heterodimerizes with a polypeptide chain signal transducer, glycoprotein 130 (GP130). The binding of IL-6 to its receptor activates several signaling pathways, including the MAPK, STAT1 and STAT3 pathways 17, 21. Activation of STAT3 can regulate the transcription of multiple downstream genes and functions in cancer metastasis 17, 22, 23.

Never in mitosis gene A (NIMA)-related kinase 9 (NEK9) is a member of the NEK family of serine/threonine kinases that are emerging as important regulators of the cell cycle and checkpoint control 24. The kinase activity is the core function of NEK9 24, 25. NEK9 regulates centrosome separation and spindle assembly during mitotic progression by phosphorylating NEK6 and NEK7, indicating its potential role in the regulation of the cytoskeleton. Currently, little is known about the roles of NEK9 in cancer 26-31. According to a very limited number of studies, NEK9 appears to be functionally associated with the MAPK/ERK pathways. In lung cancer, coexpression of NEK9 and mutant p53 was shown to promote cell proliferation by upregulating MAPK14 29. In triple-negative breast cancer, knockdown of NEK9 apparently reduces the baseline and feedback MAPK/MEK signaling and synthetic lethality in combination with PI3K inhibitors 30. NEK9-mediated phosphorylation of the microtubule-associated LC3B protein was shown to be involved in cancer metastasis and cytoskeleton-associated events 31. Additionally, NEK9 was recently reported to be recruited to the microtubules and is activated by EML4-ALK to perturb cell morphology and accelerate the migration in a microtubule-dependent manner that requires the downstream kinase NEK7 32. These findings suggest that NEK9 may participate in cancer metastasis; however, specific effects of NEK9 on aberrant motility and cytoskeletal reorganization of cancer cells remain unclear.

In the present study, we demonstrated that NEK9 can directly influence cell motility and cytoskeletal reorganization in GC, NEK9 regulates GC metastasis *via* protein kinase activity that targets the phosphorylation of ARHGEF2. Additionally, NEK9 serves as an effector of inflammation-associated signaling during GC metastasis. The activation of STAT3 by IL-6 transcriptionally suppresses miR-520f-3p and diminishes the inhibitory effects of miR-520f-3p on NEK9 and GP130. An increase in GP130, an important component of the IL-6 receptor complex, enhances the signal transduction of IL-6 stimuli and results in amplified IL-6-induced NEK9 expression. A simultaneous elevation of NEK9, GP130 and p-STAT3 in the lymph nodes and distant metastases confirms the roles of the factors in GC metastasis. Thus, we identified a novel IL-6-STAT3-NEK9 pathway that plays important roles in metastasis and cytoskeletal reorganization in GC.

## Methods

### Study approval

Inclusion of the human samples in the tissue array was approved by Xijing Hospital review board; all procedures involving animals were reviewed, and the protocols were approved by the Fourth Military Medical University Animal Care and Use Committee (KY20173286-1, approved on 03/07/2017).

### Cell lines

Human gastric cancer cell lines AGS, MKN28 and MKN45 were obtained from China Infrastructure of Cell Line Resources in September 2013. BGC823, SGC7901, SGC7901M and SGC7901NM cells were maintained in our laboratory. All cell lines were maintained in RPMI 1640 medium with 10% foetal bovine serum (South America origin; IC-1905; BioCytoSci, TX, USA) and 1% penicillin-streptomycin at 37 °C in a humidified atmosphere containing 5% CO_2_.

### Transfection, oligonucleotides and plasmids

The mimics, inhibitor and negative controls for hsa-miR-520f-3p and siRNA for ARHGEF2 were purchased from GenePharma (Shanghai, China). The shSTAT3 and shGP130 plasmids and the lentiviral vectors (full-length constructs and shRNAs) were obtained from GeneChem (Shanghai, China). The sequences are listed in Supplementary Table S1. The oligonucleotides and plasmids were transfected using Lipofectamine™ 2000 (Invitrogen, Carlsbad, CA, USA) according to the manufacturer's instructions.

### RNA extraction and quantitative real-time PCR (qRT-PCR)

Total RNA was extracted using TRIzol (Invitrogen, USA), and reverse transcription was performed using an Advantage RT-for-PCR kit (Takara Biotechnology, Japan) according to the manufacturer's instructions. Real-time PCR was conducted using SYBR Premix Pro Taq HS qPCR kit (Accurate Biotechnology, Hunan, China) and a CFX96™ real-time PCR detection system (Bio-Rad Laboratories, USA). The 2-ΔΔCt method was used to analyse the relative expression levels. The primer sequences are listed in Supplementary Table S2.

### Wound healing assay

Cells were initially cultured to full confluence in 6-well plates. Then, the cell monolayers were scratched using a 200 μL micropipette tip in the centre of the well. Then, the cells were incubated in the serum-free medium. Representative images were captured at 0 and 24 h after the injury. The width of wound healing region was quantified and compared with the baseline values by ImageJ. All experiments were repeated in triplicate independently.

### Transwell assay

A total of 4×10^4^ cells of each group in 200 μL serum-free medium were seeded into the upper chamber (8.0 μM pores, Corning, USA) without (migration) or with (invasion) Matrigel (BD Bioscience, USA). RPMI 1640 medium (600 μL) with 20% FBS was placed in the lower chamber. After incubation for 24 h, the cells in the upper chambers were fixed using 4% polymethanol and stained with crystal violet. The assay was performed in triplicate. The images were captured at 200X magnification by an Olympus BX51 microscope. Five random fields were selected, and the number of cells was counted to evaluate migration or invasion.

### Immunohistochemical analysis (IHC)

In brief, the sections were dewaxed in xylene and rehydrated in ethanol and PBS. Endogenous peroxidase was inactivated using 3% H_2_O_2_, and antigen retrieval was performed using Tris-EDTA antigen retrieval buffer for pSTAT3 staining and citrate antigen retrieval solution for NEK9 and GP130 staining. Sections were blocked with 10% normal goat serum at room temperature for 15 min and incubated with primary antibodies against pSTAT3 (#9145, Cell Signaling Technology Inc), NEK9 (NBP2-31091, NOVUS) or GP130 (PA1745, AntiProtech Inc) at 4 °C overnight. Then, the sections were incubated with the corresponding secondary antibodies conjugated with horseradish peroxidase at room temperature for 30 min. The staining was visualized using a DAB kit (Zhong Shan Golden Bridge Biotechnology, Beijing, China). All sections were examined and scored independently by two investigators in a double-blinded manner. Staining and the score were determined as described previously 33.

### Luciferase reporter assay

The plasmids (pGL3-Firefly Renilla containing miR-520f-3p sequence and mutant sequence, pGL3-Firefly Renilla containing NEK9 and GP130 3′-UTR sequence and mutant sequence) were synthesized by GeneChem (Shanghai, China). Luciferase activity was detected using a dual luciferase assay kit (GeneCopoeia) according to manufacturer's instructions. All experiments were repeated independently in triplicate.

### In vitro kinase assay

The phosphorylation of the human ARHGEF2 recombinant protein (P01) (H00009181-P01, Abnova, Tai Wan, China) by active NEK9 protein (ab125614, Abcam) was monitored using a universal kinase activity kit (R&D Systems). The amount of phosphorylated ARHGEF2 was determined as described previously 34. All experiments were repeated independently in triplicate.

### Immunoprecipitation (IP)

A/G-Agarose (Santa Cruz Biotechnology) and primary anti-IgG (B900610, Proteintech, Wuhan, China), anti-ARHGEF2 (ab155785, Abcam) and anti-NEK9 (sc-100401, Santa Cruz Biotechnology Inc) antibodies were used for the immunoprecipitation assay according to the manufacturer's instructions. Then, the samples were evaluated by western blot assay 33.

### Chromatin immunoprecipitation (ChIP)

Chromatin immunoprecipitation (ChIP) analysis was performed according to a standard method using a Magna ChIP G assay kit (EMD Millipore, USA). Chromatin was immunoprecipitated with anti-STAT3 (#12640, Cell Signaling Technology Inc), anti-IgG and in the absence of antibodies as a negative control. Immunoprecipitated DNA-protein complexes were isolated, and a qPCR assay was carried out to determine the levels of specific proteins. The primer sequences are listed in Supplementary Table S3.

### Animal experiments

BALB/C nude mice (6-week-old) were obtained from Vital River Laboratories (Beijing, China). Cells (3×10^6^) were injected into the tail veins of the nude mice to generate a metastatic model. After 8 weeks, mice were intraperitoneally injected with D-luciferin (Caliper Life Sciences). 5 min after the injection, the mice were anaesthetised, and whole-body live images were captured using an IVIS imaging system (Caliper Life Sciences). Strong signals in the lung and liver indicated metastatic tumor; thus, the mice were immediately sacrificed, and lung and liver were harvested for the detection of bioluminescence signals and final validation by haematoxylin and eosin (H&E) staining.

### Statistical analysis

The GraphPad Prism statistical package was used for statistical analyses. All data are expressed as the mean ± SD. The differences between two groups were assessed using Student *t* tests, and ANOVA test was used to compare multiple groups. The survival curves were evaluated using the Kaplan-Meier method. The Pearson and Spearman correlation coefficients were calculated (r, *p*). *P <* 0.05 was considered statistically significant.

## Results

**Figure 1:**
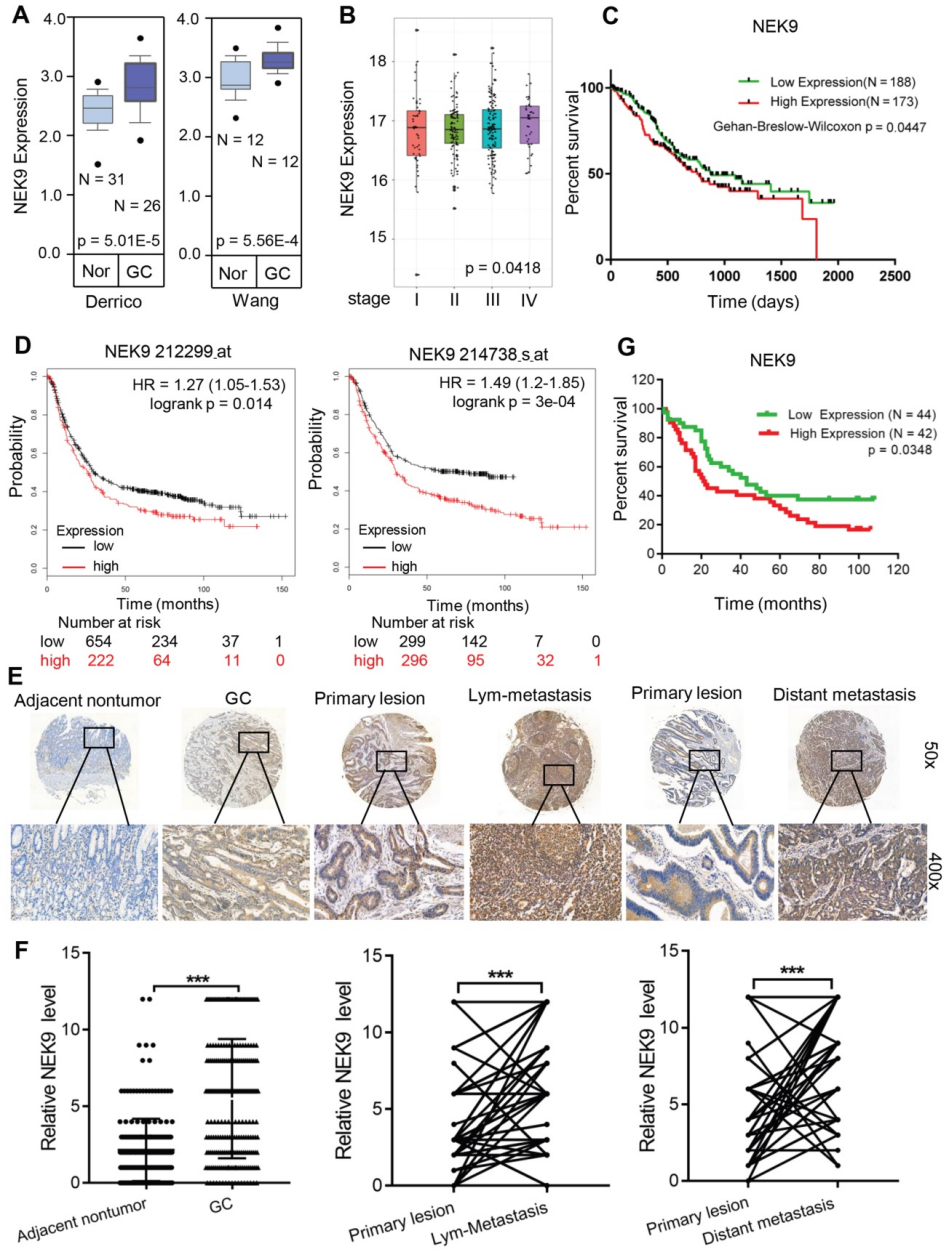
NEK9 was increased in primary GC and was correlated with cancer metastases and overall survival periods in patients with GC. A, NEK9 was increased in 2 cohorts of GC patients from Oncomine database. B-D, NEK9 was associated with advanced tumor stage (B, data from TCGA) and reduced overall survival periods (C, data from TCGA. D, data from Kaplan-Meier plotter). E-F, NEK9 was examined in multiple tissue microarrays by IHC and it was increased in primary GC, lymph node metastases and distant metastases. ^***^P < 0.001. G. An increase in NEK9 was correlated with poor prognosis of patients with GC.

**NEK9 is upregulated in GC tissues and correlates with disease progression.** To determine the role of NEK9 in GC development and progression, the NEK9 data were retrieved from the GC databases. Data analysis using ONCOMINE showed that NEK9 is upregulated in GC (Figure 1A). Then, the data from TCGA and Kaplan-Meier plotter were analysed, and the results indicated higher levels of NEK9 in advanced GC that are associated with reduced overall survival (Figures 1B-D).

NEK9 was also assayed in a cohort of 363 GC patients by IHC. The level of NEK9 was significantly increased in GC compared to that in the adjacent noncancerous tissues (Figures 1E-F). The levels were also correlated with GC progression, and higher levels of NEK9 were associated with advanced TNM staging (Table S4). In primary GC and the corresponding lymph node metastases, the staining intensity of NEK9 was significantly higher in the lymph node metastases (Figures 1E-F). A significant increase in NEK9 was also detected in the distant metastases (Figures 1E-F). Survival analysis of 86 patients with long-term follow-up showed that an increase in NEK9 is associated with reduced overall survival (Figure 1G).

**Figure 2:**
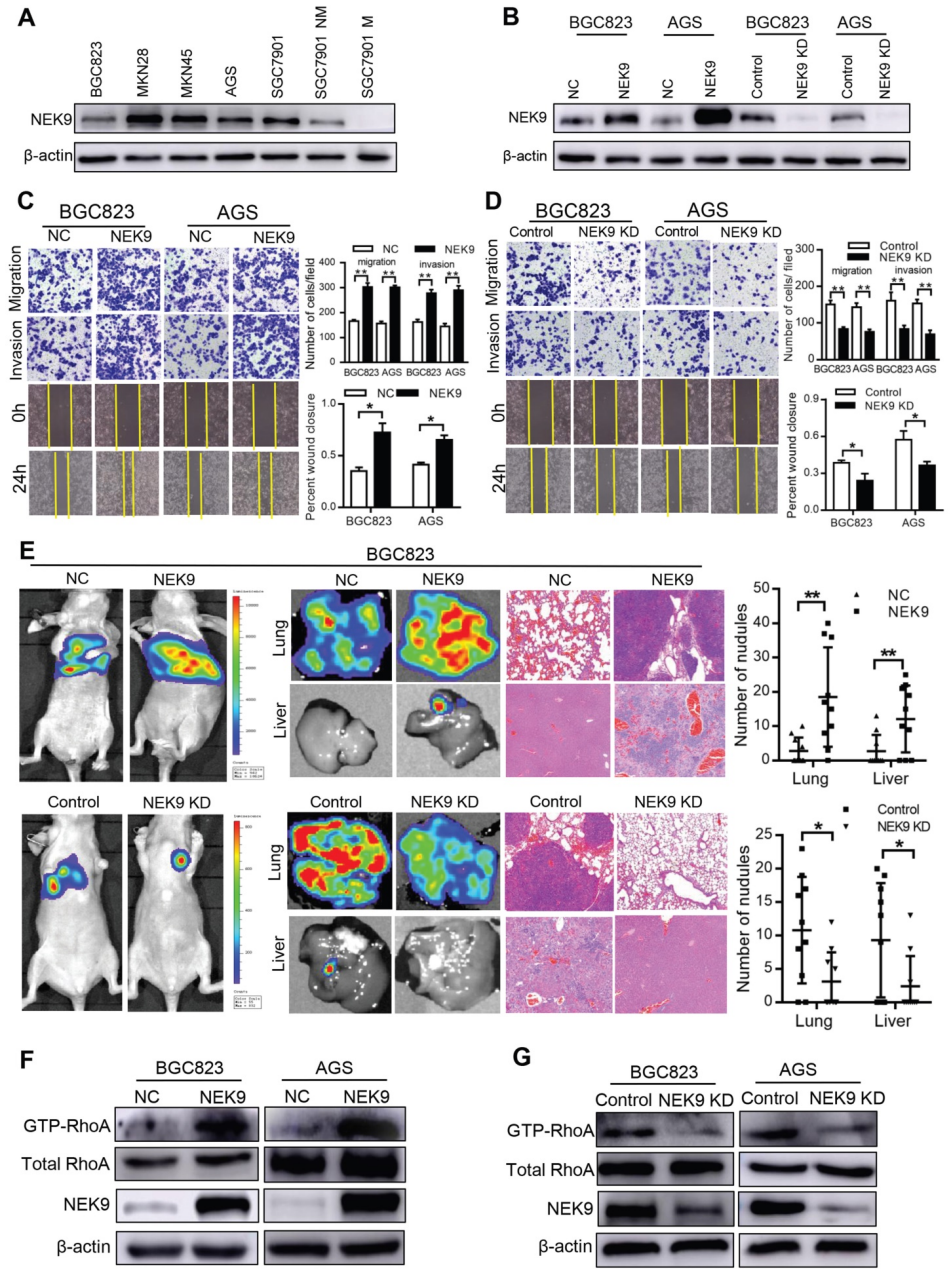
NEK9 regulated cell motility in vitro and in vivo. A-B, NEK9 was examined in multiple GC cells (A). Cell models with stable NEK9 transfection were established and validated by western blotting and RT-PCR (B). C-D, NEK9 promoted cell migration, invasion and wound healing process (D), while its knockdown inhibited cell movement (D). ^*^P < 0.05, ^**^P < 0.01. E, Ectopic NEK9 promoted cancer metastasis in vivo, and knockdown of NEK9 suppressed cancer metastasis. ^*^P < 0.05, ^**^P < 0.01. F-G, Ectopic NEK9 promoted the activation of RhoA, manifested as increased GTP-RhoA. Inhibition of NEK9 led to a decrease in GTP-RhoA.

**NEK9 regulates cell motility and cytoskeletal reorganization in GC.** BGC823 and AGS cells were used to establish cell models with stable NEK9 transfection or knockdown (Figures 2A-B). In the transwell assays, ectopic NEK9 significantly increased the number of cells migrating to the bottom side of the membrane, and NEK9 inhibition impaired the migration and invasion potential of the cells (Figures 2C-D). Similar results were obtained in the wound healing assays (Figures 2C-D). NEK9 promoted lung and liver metastases in vivo, and metastases were inhibited by NEK9 knockdown (Figure 2E).

Cytoskeletal reorganization is the basis for cell movement, and this process is precisely regulated by Rho-GTPases. A significant increase in GTP-RhoA was detected in GC cells with ectopic NEK9 (Figure 2F), indicating a transition from inactive RhoA to active RhoA. Inversely, inhibition of NEK9 decreased the level of GTP-RhoA (Figure 2G).

**Figure 3:**
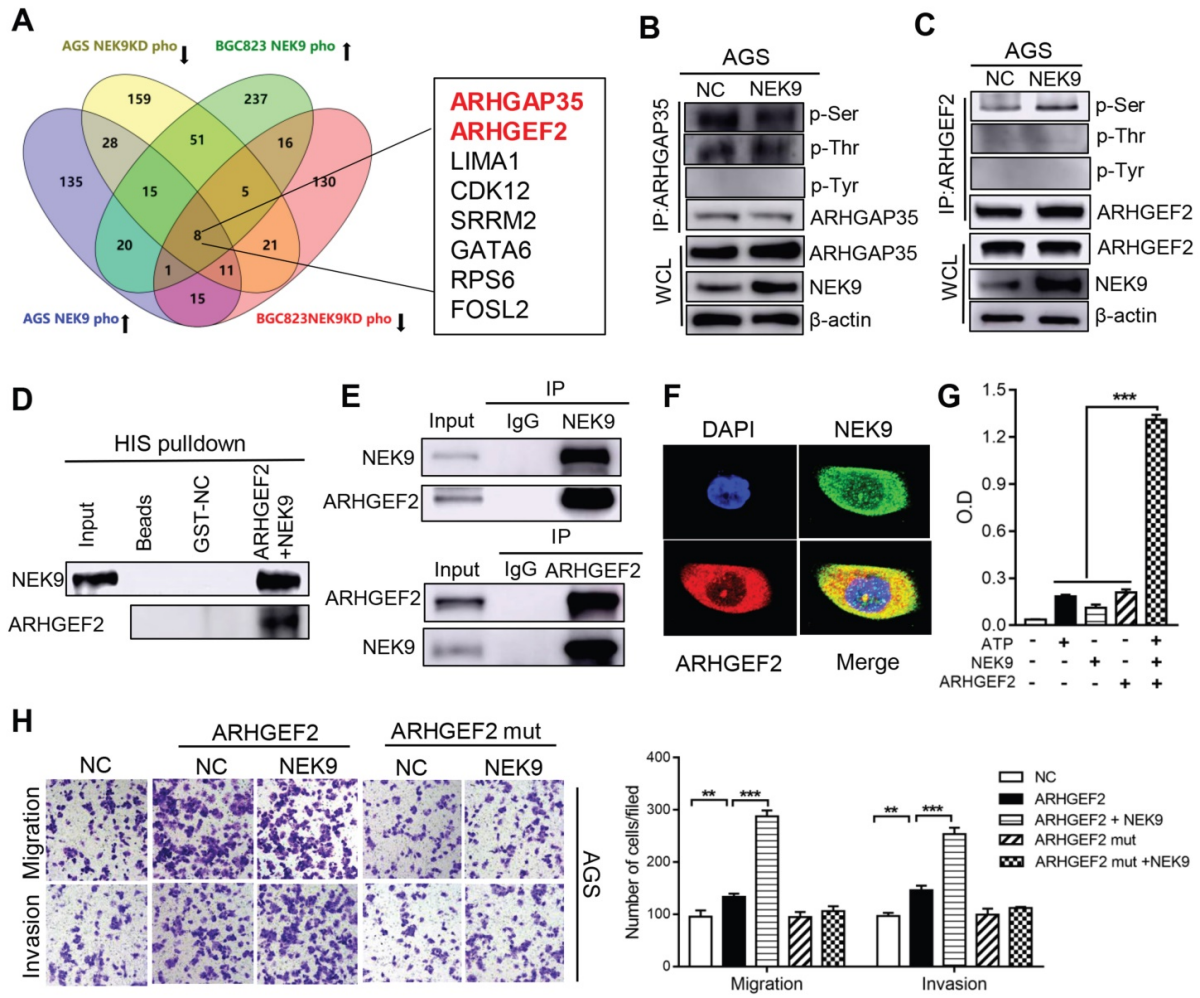
NEK9 promoted cell motility by directly phosphorylating ARHGEF2. A, The screening strategy of potential targets of NEK9. B-C, The total phosphorylation levels on serine, threonine and tyrosine of ARHGAP35 (B) and ARHGEF2 (C) were examined. D-F, The direct interaction between NEK9 and ARHGEF2 was validated by pulldown assay (D) and IP (E), and their colocalization was confirmed by immunofluorescence (F). G, NEK9 was found to directly phosphorylate ARHGEF2 by in vitro kinase assay. ^***^P < 0.001. H. The function of NEK9 and ARHGEF2 on cell motility was attenuated by mutations on all potential targeted serine residues. ^**^P < 0.01, ^***^P < 0.001.

**NEK9 regulates cell motility by targeting phosphorylation of ARHGEF2.** The intrinsic kinase activity is the key part of NEK9 function; thus, AGS and BGC823 cells with stable NEK9 transfection or knockdown were used in phosphoproteomics analyses. The ideal effectors should have increased phosphorylation in the presence of NEK9 and reduced phosphorylation when NEK9 is knocked down. A total of 8 proteins met this criterion in the AGS and BGC823 cells and were considered potential targets of NEK9 (Figure 3A). We focused our studies on ARHGEF2 and ARHGAP35 because these proteins are known to be involved in the activation of Rho GTPases. ARHGEF2 and ARHGAP35 were enriched and total phosphorylation levels on serine, threonine and tyrosine were assayed (Figures 3B-C, S1A-B). The results indicated that a specific increase in serine phosphorylation of ARHGEF2 was detected in the presence of NEK9; thus, ARHGEF2 was selected for subsequent investigation. The levels of ARHGEF2 in GC cells were manipulated to induce up- or downregulation (Figures S2A-D), and the results indicated that ARHGEF2 promotes cell motility and RhoA activation, and ARHGEF2 knockdown results in decreased cell movement and RhoA deactivation (Figures S2E-G). The binding of a kinase to a substrate is essential for phosphorylation; hence, we examined the binding of NEK9 to ARHGEF2 by pull-down assay and IP, and the results confirmed interaction of these proteins (Figures 3D-E, S1C). Colocalization of the proteins was further validated by immunofluorescent staining (Figure 3F, S1D). To investigate whether NEK9 can directly phosphorylate ARHGEF2, an in vitro kinase assay was performed, and the results indicated that NEK9 increased ARHGEF2 phosphorylation (Figure 3G), suggesting that NEK9 is a kinase that directly phosphorylates ARHGEF2.

**Figure 4:**
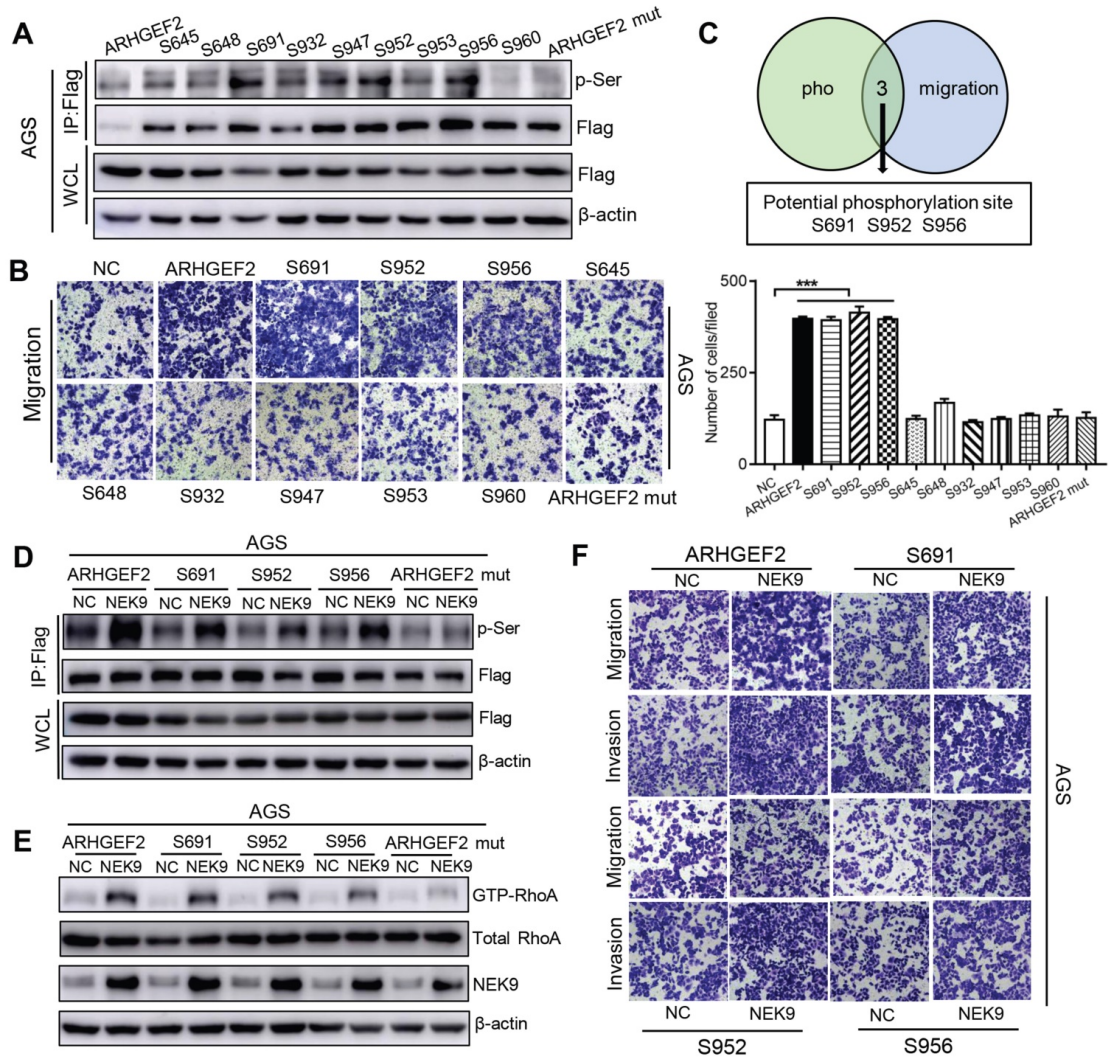
S691, S952 and S956 were the direct and functional serine residues targeted by NEK9. A-C, The phosphorylation on target serine residue was detected (A) and their roles in regulating cell motility was examined (B). The serine residues that met the requirements from A and B were listed as potential targeted serine targeted by NEK9 (C). ^***^P < 0.001. D, The phosphorylation on target serine residue of ARHGEF2 was increased at the presence of NEK9. E-F, NEK9 enhanced the effects of ARHGEF2 with wide-type S691, S952 and S956 on RhoA activation (E) and cell motility (F).

Overall, NEK9 can be a direct kinase that promotes the phosphorylation of ARHGEF2; however, the location of serine residues targeted by NEK9 was unknown. Phosphoproteomics and bioinformatics analysis identified 9 potential serine residues (Table S5). A series of ARHGEF2 plasmids was constructed, including wide-type, mutants (all potential sites were mutated) and single site wide-type plasmids (only the target site was preserved as wide-type and other 8 sites were mutated for each anticipated serine residue). The effects of ARHGEF2 were strongly attenuated when all potential serine residues were mutated (Figures 3H, S1E), suggesting that serine phosphorylation is critical to ARHGEF2 function. Additionally, the effects of NEK9 on cell motility were blocked by mutants of ARHGEF2 (Figures 3H, S1E), indicating that ARHGEF2 phosphorylation is a key step in NEK9-mediated metastasis. Then, wide-type, mutant and single site wide-type ARHGEF2 constructs were separately transfected into AGS cells, and phosphorylation of S645, S648, S691, S932, S947, S952, S953, S956 and S960 was detected (Figure 4A). However, only 3 single-site, wide-type ARHGEF2 mutants (corresponding to S691, S952 and S956) had the effects on cell motility similar to those of wide-type ARHGEF2 (Figure 4B). Therefore, S691, S952 and S956 were considered potential functional serine residue of ARHGEF2 (Figures 4C). Furthermore, increased phosphorylation of S691, S952 and S956, cell motility and GTP-RhoA were detected in the presence of NEK9 (Figures 4D-F). These results indicate that S691, S952 and S956 are the functional serine residues of ARHGEF2 phosphorylated in an NEK9-dependent manner.

**Figure 5:**
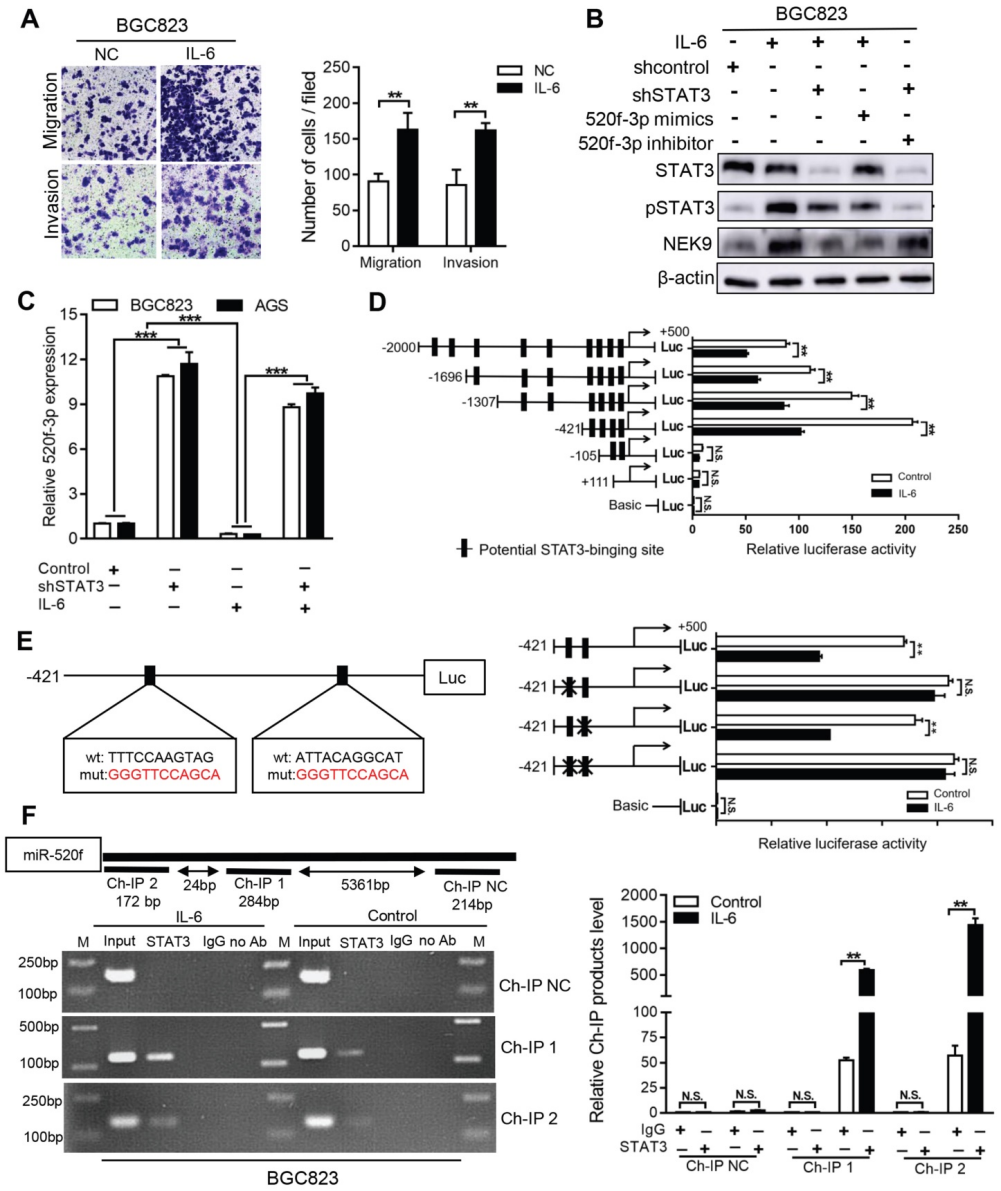
Activation of STAT3 by IL-6 directly transcriptionally suppressed miR-520f-3p. A, IL-6 increased cell migration and invasion. ^**^P < 0.01. B, IL-6 led to increased NEK9 expression through STAT3 and miR-520f-3p. C, IL-6 suppressed miR-520f-3p while knockdown of STAT3 increased miR-520f-3p. ^***^P < 0.001. D, Serially truncated and mutated miR-520f-3p promoter constructs were cloned and transfected into cells. The relative luciferase activities were determined after IL-6 stimulation.^ **^P < 0.01. E, Selective mutation (left panel) analyses identified STAT3-responsive regions in the miR-520f-3p promoter (right panel). ^**^P < 0.01. F, ChIP assay demonstrated the direct binding of STAT3 to the miR-520f-3p promoter (left panel), and the ChIP products were validated by RT-PCR (right panel).^ **^P < 0.01.

**NEK9 functions as an effector of IL-6/STAT3 inflammation signaling *via* miR-520f-3p.** Inflammatory stimuli are known to participate in cancer metastasis 17-20. Stimulation of GC cells with IL-6 increased cell motility and level of NEK9 (Figures 5A-B). Knockdown of STAT3 blocked the effect of IL-6 on NEK9 (Figure 5B), showing that IL-6-induced increase in NEK9 is mediated by activation of STAT3. In our previous study, miR-520d was shown to be affected by the IL-6/STAT3 signaling 20. Therefore, we used multiple independent databases to computationally predict miRNAs that may be involved in the process. miR-520f-3p was one of the candidate miRNAs that attracted our attention. The mimics or inhibitors of miR-520f-3p were transfected into GC cells, and the results of RT-PCR and western blot indicated that miR-520f-3p negatively regulates NEK9 (Figures S3A-D). To determine whether miR-520f-3p represses NEK9 by targeting a potential binding site, PCR products containing wild-type or mutant NEK9 3'-UTR sequences were cloned downstream of luciferase open reading frame. Exogenous miR-520f-3p suppressed the luciferase activities in the cells transfected with the NEK9 3'-UTR reporter constructs; however, this effect was abolished when the seed sequences in the constructs were mutated (Figure S3E). Furthermore, the inhibitory effect of miR-520f-3p on cell motility was partially attenuated by ectopic NEK9 expression (Figure S3F). Conversely, knockdown of NEK9 antagonized the effect of miR-520f-3p inhibitors (Figure S3G).

IL-6 suppressed the levels of miR-520f-3p, and knockdown of STAT3 abolished this effect (Figure 5C). Ectopic expression of miR-520f-3p inhibited the IL-6-mediated increase in NEK9, and a miR-520f-3p inhibitor reversed the suppressive effects of STAT3 knockdown on NEK9 (Figure 5B). Then, we determined whether IL-6/STAT3 could upregulate NEK9 by inhibiting miR-520f-3p. To validate our hypothesis, a series of miR-520f-3p promoter truncation mutants was generated targeting the potential binding sites of STAT3 on miR-520f-3p promoter predicted by Jaspar (Tables S6-7). The results of the luciferase assay after IL-6 treatment showed that the regulatory region may be located between -421 and -105 bp (Figure 5D). Two potential binding sites were present in this region, and single- or double-site mutant promoters were constructed (Table S8, Figure 5E). No significant differences in the effects of the wide-type and site-2 mutant sequences were observed; however, site-1 mutation was sufficient to block the binding activity (Figure 5E). Consistently, ChIP analysis showed that IL-6 treatment significantly increased the association of STAT3 with the miR-520f-3p promoter (Figure 5F). Overall, these results indicate that the IL-6/STAT3 pathway suppresses miR-520f-3p transcription in GC cells.

**Figure 6:**
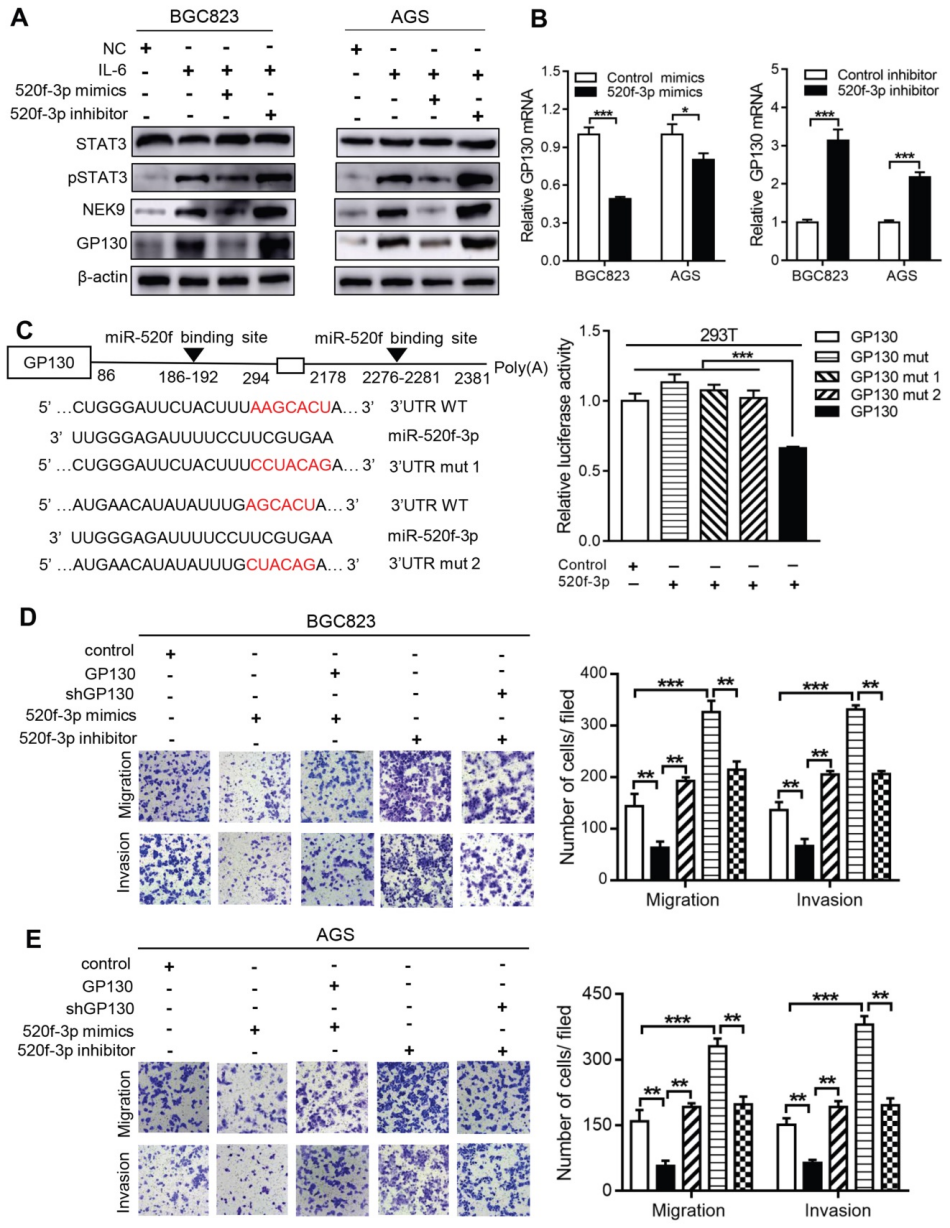
miR-520f-3p modulated STAT3 phosphorylation through GP130. A, IL-6 stimulation led to increased STAT3 phosphorylation, NEK9 and GP130, while miR-520f-3p antagonized these effects. ^**^P < 0.01,^ ***^P < 0.001. B, miR-520f-3p suppressed GP130 mRNA while its inhibition increased GP130 mRNA. ^*^P < 0.05,^ ***^P < 0.001. C, Mutations were generated at the predicted miR-520f-3p binding sites in the 3'UTR of GP130 (left panel), and luciferase reporter assay showed that miR-520f-3p directly bind to 3'UTR of GP130 (right panel). ^***^P < 0.001. D-E, The inhibitory effect of miR-520f-3p on cell motility was blocked by ectopic GP130. Inhibition of miR-520f-3p promoted cell motility and GP130 knockdown antagonized this effect. ^**^P < 0.01,^ ***^P < 0.001.

**MiR-520f-3p targets GP130 and inhibits STAT3 activation caused by IL-6.** The activation of STAT3 by IL-6 can transcriptionally suppress miR-520f-3p to increase NEK9. Interestingly, miR-520f-3p was able to inhibit STAT3 activation in the presence of IL-6 (Figure 6A). This effect suggested a feedback between IL-6/STAT3 and miR-520f-3p. To validate this hypothesis, we reviewed the potential targets of miR-520f-3p and identified GP130, an important part of the IL-6 receptor complex, as a possible effector of miR-520f-3p in the IL-6/STAT3 signaling. The suppression of GP130 by miR-520f-3p was detected by western blot and RT-PCR (Figures 6A-B), and inhibitors of miR-520f-3p increased the level of GP130 (Figure 6B). The luciferase reporter gene assays showed that miR-520f-3p can directly bind to the 3'-UTR of GP130 (Figure 6C). Furthermore, the inhibition of cell motility by miR-520f-3p was attenuated by ectopic expression of GP130 (Figures 6D-E). Conversely, knockdown of GP130 antagonized the effect of miR-520f-3p inhibitors (Figures 6D-E). Thus, a feedback pathway of GC metastasis involving IL-6/STAT3/NEK9 was identified and validated.

**Figure 7:**
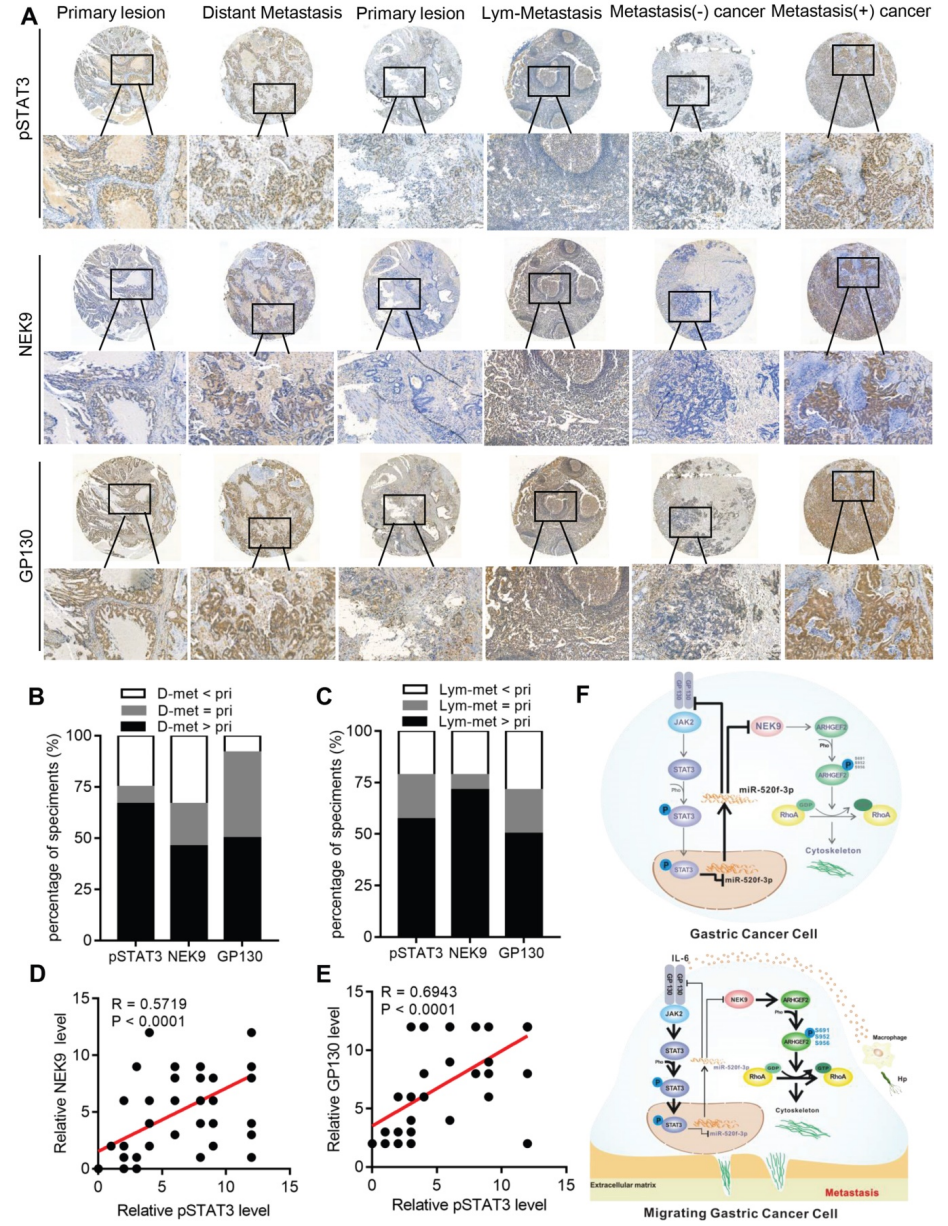
A simultaneous elevation of p-STAT3, NEK9 and GP130 was found in metastatic GC, lymph nodes and distant metastases. A-C, p-STAT3, NEK9 and GP130 in lymph nodes and distant metastases were examined by IHC (A), and an increase in these proteins was found in cancer metastases (B-C). D-E, The positive correlation of p-STAT3, NEK9 and GP130 was found in GC. F, Schematic model of GC metastasis. IL-6 induced by inflammation from immunological, infectious and chemical factors activated STAT3 which directly suppressed the expression of miR-520d-5p, causing increased NEK9 and GP130. An increase in GP130 enhances this IL-6-STAT3 signaling, and NEK9 directly affects cell motility and cytoskeleton reorganization by targeting the phosphorylation of ARHGEF2.

**p-STAT3, NEK9 and GP130 are simultaneously upregulated in GC metastases and correlate with poor prognosis in GC patients.** To evaluate the clinical significance of p-STAT3, NEK9 and GP130 in GC patients, the expression of p-STAT3, NEK9 and GP130 was analysed in 2 sets of GC tissue microarrays *via* IHC. The results obtained in primary GC and the corresponding lymph node metastases indicated that the staining intensity of p-STAT3, NEK9 and GP130 was significantly higher in the lymph node metastases (Figures 7A-7B). Comparison of primary GC and the corresponding distant metastases indicated that the staining intensity was significantly higher in the distant metastases (Figures 7A, 7C). Compared of the expression levels in nonmetastatic primary GC tissues indicated that p-STAT3, NEK9 and GP130 levels are increased in the metastatic primary GC tissues (Figure 7A). Additionally, a positive correlation between p-STAT3, NEK9 and GP130 was detected (Figures 7D-E) in agreement with the results obtained using the data of the TCGA database (Figures S4A-B). Analysis of the data of the TCGA and Kaplan-Meier plotter databases showed that an increase in STAT3 and GP130 is associated with reduced overall survival of GC patients (Figures S4C-E). These findings suggest the potential roles of this pathway in evaluation of the metastatic potential and prognosis of GC patients.

In conclusion, we report a novel pathway in the metastasis of GC. As shown in Figure 7F, STAT3 in GC cells can be activated by the inflammatory stimuli, which directly suppress miR-520f-3p at the transcription level. Decreased miR-520f-3p induces an increase in NEK9 and GP130. Increased NEK9 promotes ARHGEF2 phosphorylation and RhoA activation to induce cytoskeleton reorganization and cell movement. The IL-6-STAT3 pathway is then further activated by an increase in GP130 to enhance GC metastasis driven by inflammation in the tumor microenvironment. The feedback axis identified in our study can serve as a potential therapeutic target of metastasis in GC.

## Discussion

In the present study, we identified an IL-6-triggered circuit involving the STAT3-mediated suppression of miR-520f-3p and the downstream targets, NEK9 and GP130. The results indicate that this pathway regulates cancer metastasis, confirming that inflammation in tumor microenvironment may serve as an important driving force in the initiation of cancer metastasis.

Chronic inflammation is known to promote the development and progression of cancer 16, 17, 35. IL-6 is produced by multiple cell types in the tumor microenvironment, including tumor-infiltrating immune cells, stromal cells and tumor cells 21. Elevated IL-6 stimulates hyperactivation of STAT3 to include the expression of the genes associated with angiogenesis, invasiveness and metastasis of cancer 21, 36. Atrophic gastritis, *H. pylori* infection, refluxed bile acids, surgery and chemoradiation are the sources of inflammation in GC 37, 38. The results of our study indicate that IL-6/STAT3 signaling induces and promotes cancer metastasis. Activation of IL-6/STAT3 suppresses miR-520f-3p transcription that amplifies the hyperactivation of STAT3 by targeting GP130. The positive feedback loop demonstrated here suggests that IL-6/STAT3 play critical roles in the initiation and promotion of cancer metastasis. Therefore, targeting this pathway may have potential therapeutic effects.

The main approaches to inhibit IL-6-mediated signaling at the ligand and/or receptor levels include targeting IL-6/IL-6R with antibodies and targeting the IL-6-sIL-6R complex using fusion proteins incorporating GP130 21. The antitumor efficacy of siltuximab, a chimeric mouse-human antibody against IL-6, against multiple cancers was demonstrated; however, this effect was absent in advanced cancers 39-41. Tocilizumab is a humanized monoclonal antibody that recognizes IL-6R. Combination of tocilizumab with chemotherapy is potentially safe and feasible 42. Selective inhibition by proteins incorporating the gp130 sequence may provide a solution to the IL-6 targeted therapy, but its safety and efficacy requires additional investigations in clinical trials^21^. Currently, a few preclinical and clinical studies investigated targeting IL-6/STAT3 in therapy in GC, and the findings of our study provide a solid theoretical support for clinical applications of anti-IL-6 strategy of treatment of GC metastasis.

Our study demonstrated that miR-520f-3p is critical for the amplification of the metastasis-promoting effects of inflammatory stimuli. A significant reduction in miR-520f-3p was detected when GC cells were stimulated with IL-6, indicating an inverse correlation between IL-6/STAT3 and miR-520f-3p. The transcriptional regulation by activated STAT3 is the key component of the IL-6/STAT3 pathway; thus, specific binding sites of STAT3 were identified in the promoter region of miR-520f-3p demonstrating that miR-520f-3p transcription is directly regulated by IL-6-medated STAT3 activation. miR-520f-3p suppresses GP130 by targeting its 3'-UTR, which is a critical step in the amplification of the effects of the IL-6/STAT3 pathway. In fact, the regulation of GP130 by miR-520f-3p may be a fundamental and universal molecular event in inflammation, since similar findings were reported in liver cells 43. According to the reports of the literature, miR-520f-3p is functional in liver cancer and GC 44, 45, and targeting of SOX9 by miR-520f-3p regulated the sensitivity to targeted therapy and cancer stem cell phenotype 44. Direct modification of miR-520f-3p or the upstream IL-6/STAT3 signaling induced significant alterations in the metastatic capacity of GC cells. These results suggest a new role of miR-520f-3p in cancer progression and indicate that targeting of miR-520f-3p by direct or indirect means may be a potentially promising therapeutic option in GC.

The fundamental role of NEK9 is focused on mitosis. Activated NEK9 phosphorylates NEK6 and NEK7, which subsequently phosphorylate the components (Eg5, microtubules and c-TuRC) that are necessary for proper mitotic spindle formation 24. Despite the fact that the role of NEK9 in this process involves cytoskeleton reorganization, the effects of NEK9 on cell motility were rarely investigated previously. Thus, our study is the first to systemically investigate the effects of NEK9 on cell motility, which considerably extends current understanding of the role of NEK9 in cancer.

According to the previous studies, the intrinsic kinase activity is critical to the biological function of NEK9 24, 29, 31. Phosphoproteomics analysis was used to screen for potential substrates of NEK9, and the data confirmed that NEK9 can directly phosphorylate ARHGEF2. Notably, not all phosphorylation sites were functional. The extent of phosphorylation of each functional site was highly variable. Blockade, mutation or replacement of the functional phosphorylation serine residues of ARHGEF2 is involved in the precise control of the function of NEK9, which may avoid the potential side effects of direct NEK9 silencing. Phosphorylation is an important posttranslational modification involved in the maintenance and enhancement of ARHGEF2 function 13, 14. NEK9-medidated ARHGEF2 phosphorylation contributes to increased cell movement by inducing direct transformation of inactive RhoA to the active state to enable local invasiveness and distant metastasis of cancer cells. According to a previous study, activated STAT3 regulates microtubule dynamics and releases ARHGEF2 to activate RhoA thus promoting amoeboid migration of diffuse large B-cell lymphoma 22. The results of this study suggest that in addition to the regulation by NEK9, ARHGEF2 may be directly regulated by STAT3. All these evidences suggest that the IL-6/STAT3/miR-520f-3p /NEK9/GP130 feedback loop demonstrated here is a key molecular signaling pathway in GC metastasis.

A significant increase in NEK9 was detected in lymphoid nodes and distant metastasis and was associated with higher TNM staging. The examination of clinical GC specimens indicated coincidental upregulation of p-STAT3, GP130 and NEK9. Analysis of the data of multiple online databases indicated that STAT3, GP130 and NEK9 are associated with reduced overall survival, and their levels are mutually positively correlated. The data of the clinical assessment suggest that this feedback loop has clinical significance and provide preclinical support for potential use of the key molecules of this pathway as biomarkers of metastasis and survival evaluation in GC.

## Conclusion

In summary, we demonstrated that chronic inflammation in the tumor environment can stimulate the activity of the IL-6/STAT3 signaling, and STAT3 can directly inhibit the transcription of miR-520f-3p. miR-520f-3p targets GP130 to form a closed positive feedback loop with IL-6/STAT3. NEK9, a target of miR-520f-3p, can directly influence cell motility by phosphorylating ARHGEF2. The novel IL-6/STAT3/miR-520f-3p/NEK9/GP130 feedback loop may contribute to an improved understanding of the role of inflammatory signaling in GC metastasis.

## Supplementary Material

Supplementary methods, figures and tables.

## Abbreviations

GEFs: guanine nucleotide exchange factors
GAPs: GTPase activating proteins
H&E: hematoxylin and eosin
IHC: Immunohistochemical analysis
IP: Immunoprecipitation
CHIP: chromatin immunoprecipitation assay
EDTA: Ethylene Diamine Tetraacetic Acid
DAB: Diaminobenzidine
TNM: Tumor, Lymph Node, Metastasis
