# Supplementary material for: NEK9, a novel effector of IL-6/STAT3, regulates metastasis of gastric cancer by targeting ARHGEF2 phosphorylation
Source: Theranostics. 2021 Jan 1;11(5):2460–74. doi: 10.7150/thno.53169 (PMC7797683; doi:10.7150/thno.53169)
Supplement: Supplementary file 1 — Supplementary methods, figures and tables. [file thnov11p2460s1.pdf]

Methods

Tissue microarrays

Tissue microarrays HStmA180Su15, HStm-Ade120Lym-01, HStm-Ade150CS1-01 and HStmA050Me01 were purchased from Shanghai Outdo Biotech (Outdo, Shanghai, China). Another microarray (T14-129, containing 105 gastric cancer cases) with complete pathologic were provided by Xijing Hospital of Digestive Diseases, Fourth Military Medical University (Xi'an, Shaanxi, China).

Immunofluorescence analysis (IF)

AGS cell were seeded in 4-well chamber slides, and then washed with sterile PBS and fixed with 4% paraformaldehyde for 20 min at room temperature. Then, they were permeabilized 10 min with 1% TritonX-100. After a 30min incubation in PBS containing 0.05% Tween-20 (PBST) and 3% BSA, cells were incubated primary antibody rabbit anti-ARHGEF2 (ab155785, Abcam) and mouse anti-NEK9 (Santa Cruz Biotechnology Inc) at 4 °C overnight. The next day, cells were washed in PBS three times and incubated with conjugated the secondary antibody (1:500) for 1 hour at room temperature. Immunofluorescence images were captured using a Nikon A1 Plus confocal microscope.

Mass Spectrometry (MS)

Proteins were extracted from AGS and BGC823 cell lines with stable overexpressioning NEK9, knockdown NEK9 and their controls. A quantity of 150 μg of protein from each sample was digested with different enzymes, the digested peptide (100 μg) from different samples were labeled with tandem mass tags (TMT) reagents (Thermo, Pierce Biotechnology, USA). Phosphopeptide enrichment was performed on 5 μM titansphere bulk particles (Canadian Life Science, Peterborough, ON) according to manufacturer's protocols. The data analyzed by nano-LC-MS/MS. Comprehensive data mining and filtering identified a total of 8 crossover proteins in different proteins form each sample, 2 of which

were related to cytoskeleton.

In vitro pull-down assay

Purified ARHGEF2 was mixed with anti-ARHGEF2 antibody and agarose G for 4 h and washed three times with 50 mM Tris-HCl (pH 7.4) binding buffer containing 100 mM NaCl, 0.5% Triton X-100, 10% glycerol, 1 mM EDTA, and protease inhibitor (Calbiochem) at 4 °C. The antibody conjugated ARHGEF2 proteins were incubated with NEK9 proteins. After 12 h, the coprecipitates with ARHGEF2 were harvested by centrifugation at 5000 rpm and washed three times with binding buffer at 4 °C. The NEK9 and ARHGEF2 in precipitates were detected by SDS-PAGE and western blotting. The purified ARHGEF2 proteins were mixed with cell lysates in 50 mM Tris-HCl (pH 7.4) binding buffer containing 100 mM NaCl, 0.5% Triton X100, 10% glycerol, 1 mM EDTA, and protease inhibitor (Calbiochem) at 4 °C overnight. The proteins were precipitated by glutathione-Sepharose beads and washed with binding buffer three times, and the coprecipitates were separated by SDS-PAGE. The amounts of NEK9 and ARHGEF2 proteins were detected by western blotting.

Supplemental figures

Figure S1

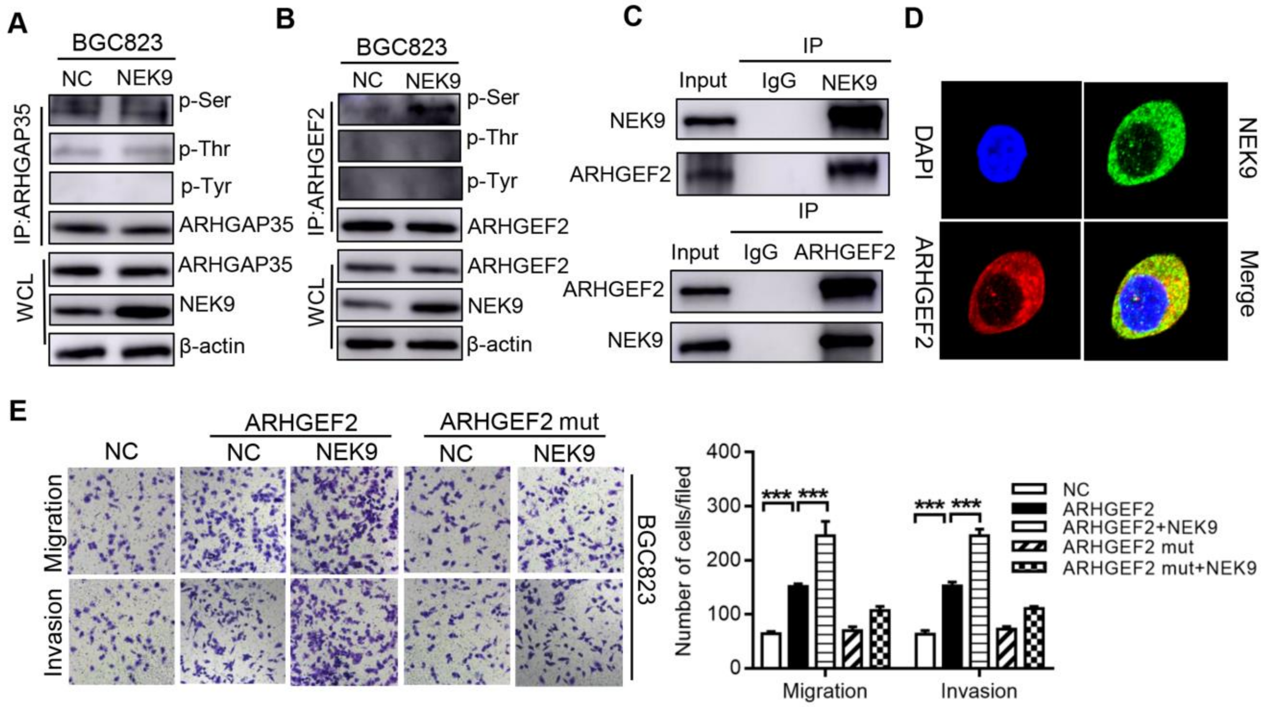

Figure S1.

NEK9 interacted with ARHGEF2 and promoted cell motility by through phosphorylating ARHGEF2. A-B, The total phosphorylation levels on serine, threonine and tyrosine of ARHGAP35 (A) and ARHGEF2 (B) were examined. C-D, The direct interaction between NEK9 and ARHGEF2 was validated by IP (C), and their colocalization was confirmed by immunofluorescence (D). E, The function of NEK9 and ARHGEF2 on cell motility was attenuated by mutations on all potential targeted serine residues. $^{***}P<0.001$.

Figure S2

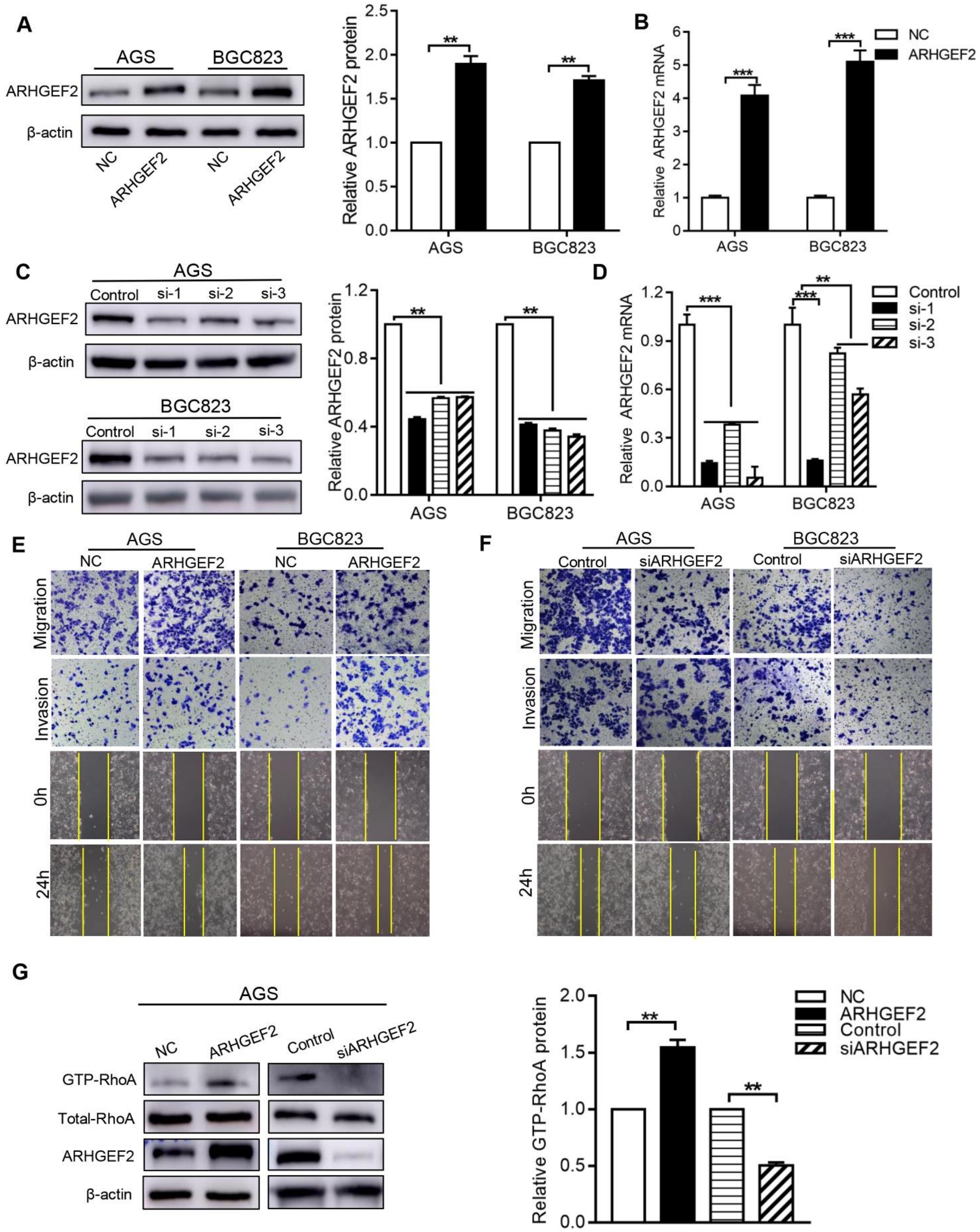
A
AGS
BGC823
ARHGEF2
β-actin
NC
ARHGEF2
NC
ARHGEF2
Relative ARHGEF2 protein
AGS
BGC823
B
Relative ARHGEF2 mRNA
NC
ARHGEF2
AGS
BGC823
C
AGS
Control
si-1
si-2
si-3
ARHGEF2
β-actin
BGC823
Control
si-1
si-2
si-3
ARHGEF2
β-actin
Relative ARHGEF2 protein
AGS
BGC823
D
Relative ARHGEF2 mRNA
Control
si-1
si-2
si-3
AGS
BGC823
E
AGS
BGC823
NC
ARHGEF2
NC
ARHGEF2
Migration
Invasion
0h
24h
F
AGS
BGC823
Control
siARHGEF2
Control
siARHGEF2
Migration
Invasion
0h
24h
G
AGS
NC
ARHGEF2
Control
siARHGEF2
GTP-RhoA
Total-RhoA
ARHGEF2
β-actin
Relative GTP-RhoA protein
NC
ARHGEF2
Control
siARHGEF2

Figure S2.

ARHGEF2 regulated cell motility and cytoskeleton reorganization. A-D, ARHGEF2 was up- or down-regulated in GC cells. $^{**}P<0.01$, $^{***}P<0.001$. E-F, Ectopic ARHGEF2 promoted cell motility while its knockdown suppressed cell movement. G, ARHGEF2 promoted RhoA activation and its knockdown was sufficient to suppress RhoA activity. $^{**}P<0.01$.

Figure S3

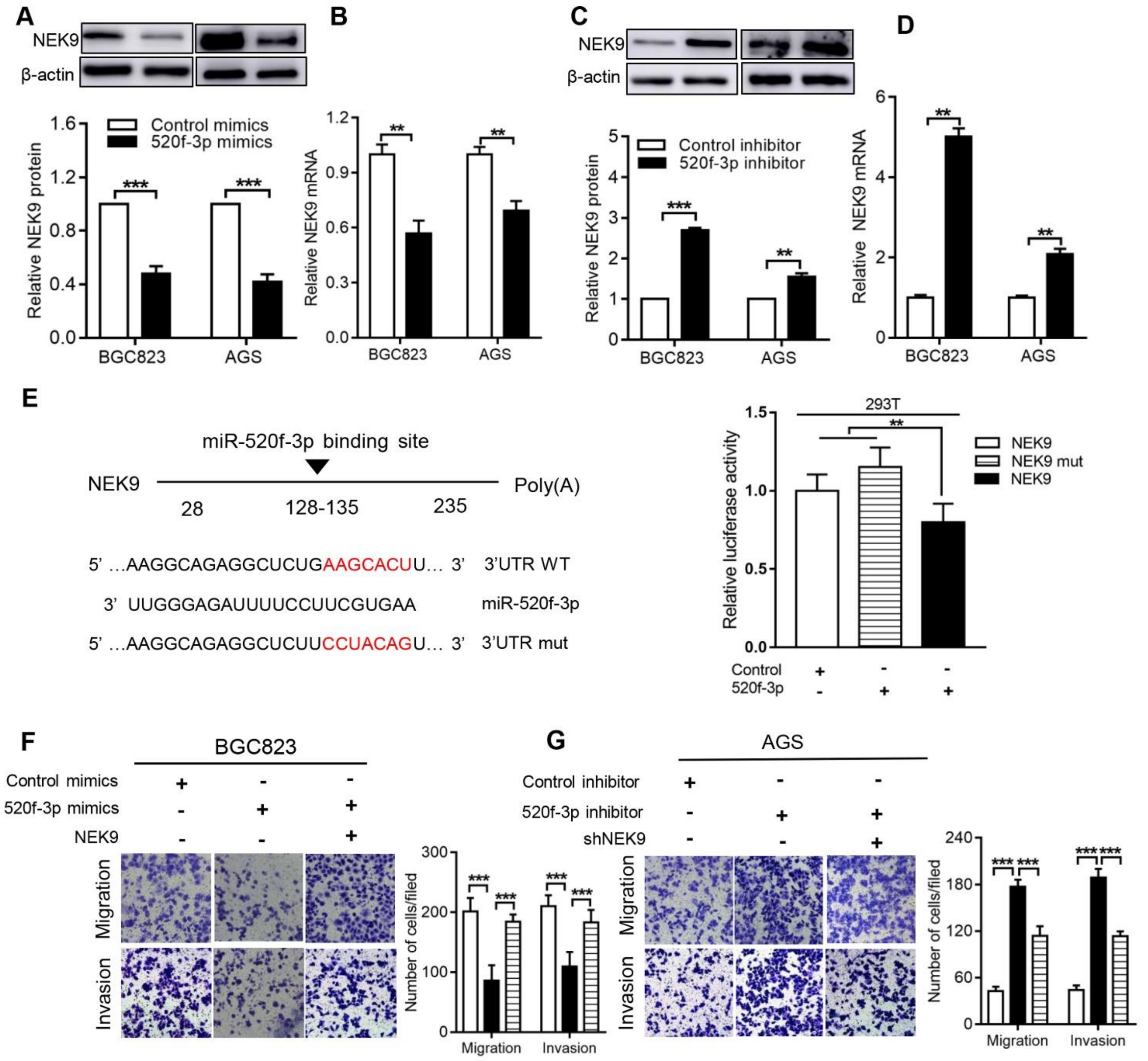
A
NEK9
β-actin
Control mimics
520f-3p mimics
Relative NEK9 protein
BGC823
AGS
B
Relative NEK9 mRNA
C
Control inhibitor
520f-3p inhibitor
D
E
miR-520f-3p binding site
NEK9
Poly(A)
28
128-135
235
5' …AAGGCAGAGGCUCUGAAGCACUU… 3'
3'UTR WT
3' UUGGGAGAUUUUCCUUCGUGAA
miR-520f-3p
5' …AAGGCAGAGGCUCUUCCUACAGU… 3'
3'UTR mut
293T
Relative luciferase activity
NEK9 mut
Control
520f-3p
F
BGC823
Control mimics
520f-3p mimics
NEK9
Migration
Invasion
Number of cells/filed
G
AGS
Control inhibitor
520f-3p inhibitor
shNEK9

Figure S3.

NEK9 was the direct target of miR-520f-3p. A-B, miR-520f-3p suppressed NEK9 at protein and mRNA levels. $^{**}P<0.01$. C-D, Inhibition of miR-520f-3p increased NEK9 at protein and mRNA levels. $^{**}P<0.01$, $^{***}P<0.001$. E, Mutations were generated at the predicted miR-520f-3p binding sites in the 3’UTR of NEK9 (left panel), and luciferase reporter assay showed that miR-520f-3p could directly bind to 3’UTR of GP130 (right panel). $^{**}P<0.01$. F, The inhibitory effect of miR-520f-3p on cell motility was blocked by ectopic NEK9. $^{***}P<0.001$. G, Inhibition of miR-520f-3p promoted cell motility and knockdown of NEK9 antagonized this effect. $^{***}P<0.001$.

Figure S4

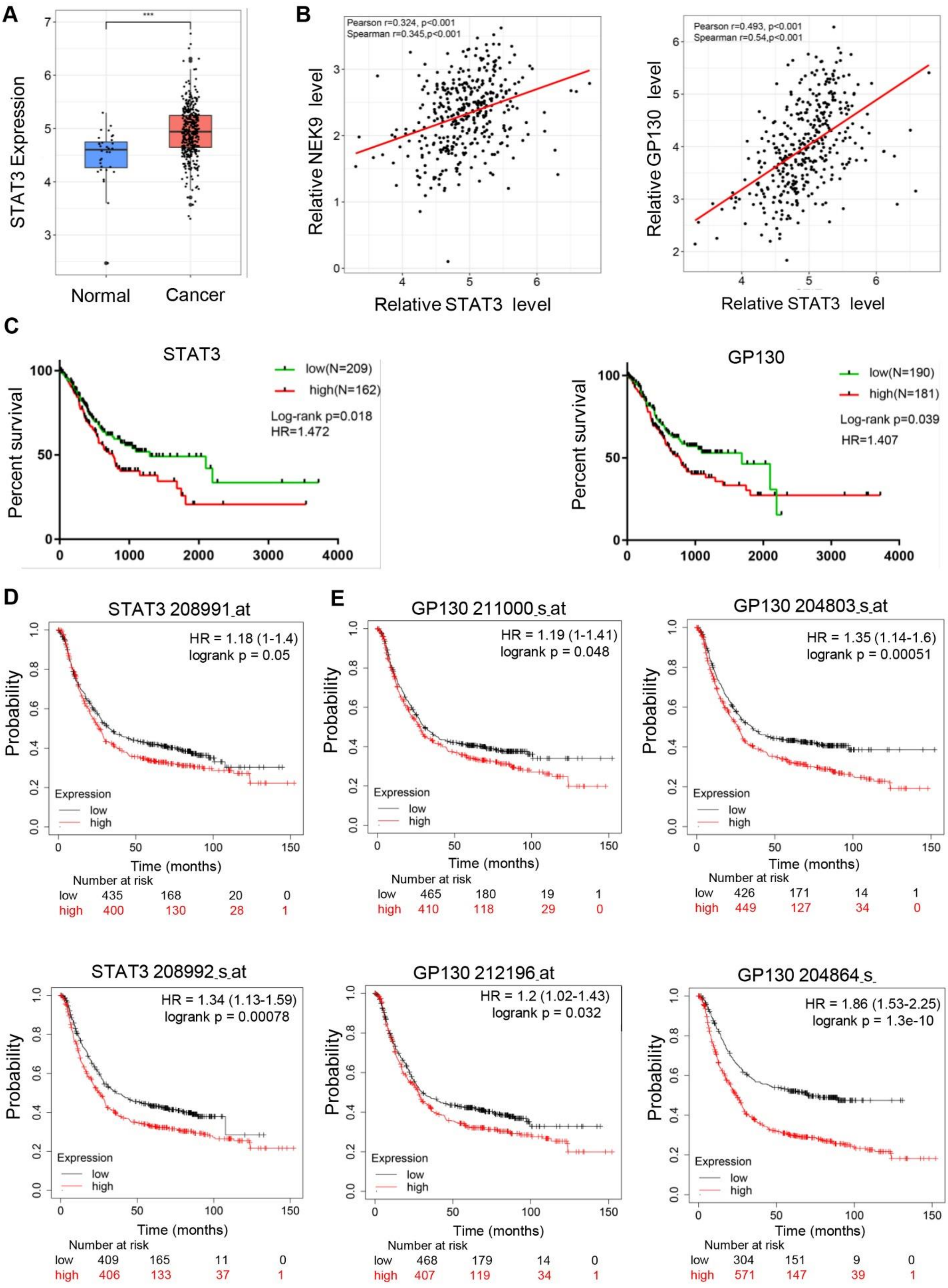
A
STAT3 Expression
***
Normal
Cancer
B
Pearson r=0.324, p<0.001
Spearman r=0.345,p<0.001
Relative NEK9 level
Relative STAT3 level
Pearson r=0.493, p<0.001
Spearman r=0.54,p<0.001
Relative GP130 level
Relative STAT3 level
C
STAT3
low(N=209)
high(N=162)
Log-rank p=0.018
HR=1.472
Percent survival
GP130
low(N=190)
high(N=181)
Log-rank p=0.039
HR=1.407
Percent survival
D
STAT3 208991_at
HR = 1.18 (1-1.4)
logrank p = 0.05
Probability
Expression
low
high
Time (months)
Number at risk
low 435 168 20 0
high 400 130 28 1
E
GP130 211000_s_at
HR = 1.19 (1-1.41)
logrank p = 0.048
Probability
Expression
low
high
Time (months)
Number at risk
low 465 180 19 1
high 410 118 29 0
GP130 204803_s_at
HR = 1.35 (1.14-1.6)
logrank p = 0.00051
Probability
Expression
low
high
Time (months)
Number at risk
low 426 171 14 1
high 449 127 34 0
STAT3 208992_s_at
HR = 1.34 (1.13-1.59)
logrank p = 0.00078
Probability
Expression
low
high
Time (months)
Number at risk
low 409 165 11 0
high 406 133 37 1
GP130 212196_at
HR = 1.2 (1.02-1.43)
logrank p = 0.032
Probability
Expression
low
high
Time (months)
Number at risk
low 468 179 14 0
high 407 119 34 1
GP130 204864_s_
HR = 1.86 (1.53-2.25)
logrank p = 1.3e-10
Probability
Expression
low
high
Time (months)
Number at risk
low 304 151 9 0
high 571 147 39 1

Figure S4.

Data analyses from database showed that the positive correlation of p-STAT3, NEK9 and GP130 was found and increased these molecules were associated with reduced overall survival of GC patients. A, STAT3 was increased in GC. B, the positive correlation of p-STAT3, NEK9 and GP130 in GC. C, Data analysis from TCGA showed that higher STAT3 and GP130 were associated with reduced overall survival. D-E, Data analysis from Kaplan-Meier plotter showed that higher STAT3 and GP130 were associated with reduced overall survival.

Supplemental Tables

Table S1. oligonucleotides and plasmids

Name	Sequences
miR-520f-3p-mimics sense:	5'-AAGUGCUUCCUUUUAGAGGGUU -3'
miR-520f-3p-mimics antisense:	5'-CCCUCUAAAAGGAAGCACUUUU -3'
miR-520f-3p-inhibitor sense:	5'-AACCCUCUAAAAGGAAGCACUU -3'
NEK9-RNAi sense	5'- CCGAGGAATGGAAGGTTTAAT-3'
STAT3-RNAi sense	5'- GCTGACCAACAATCCCAAGAA-3'
GP130-RNAi sense	5'- GCAACACACAAGTTTGCTGAT-3'
siARHGEF2-1 sense:	5'-CCCUGUACUUGAGUUUCAATT -3'
siARHGEF2-1 antisense:	5'-UUGAAACUCAAGUACAGGGTT -3'
siARHGEF2-2 sense:	5'-CCAAGUACCCGUUACUCAUTT -3'
siARHGEF2-2 antisense:	5'-AUGAGUAACGGGUACUUGGTT -3'
siARHGEF2-3 sense:	5'-GCGAUUGGUCAAUCUCUAUTT -3'
siARHGEF2-3 antisense:	5'-AUAGAGAUUGACCAAUCGCTT -3'

Table S2. Primers for real-time PCR

Name	Sequences
NEK9 sense:	5'-GCTGTGATGGGACATTTCTG -3'
NEK9 antisense:	5'-CCAAGGTAAAGGACGTTGTG -3'
ARHGEF2 sense:	5'-CAGGCATGACCATGTGCTATG -3'
ARHGEF2 antisense:	5'-TTTACAGCGGTTGTGGATAGTC -3'
GP130 sense:	5'-TCTGGGAGTGCTGTTCTGCT -3'
GP130 antisense:	5'- TGTGCCTTGGAGGAGTGTGA -3'
Actin sense:	5'-CGTACCACTGGCATCGTGAT -3'
Actin antisense:	5'-GTGTTGGCGTACAGGTCTTTG -3'
miR-520f-3p sense	5'-AAGTGCTTCCTTTTAGAGGGTT -3'

Table S3. Primers used for ChIP in the miR-520f promoter

Name	Sequences
miR-520f Ch-IP NC sense:	5'-TGCACTAATGACACCTTTGAA-3'
miR-520f Ch-IP NC antisense:	5'-AGACTGGATAGACTTGGAGGC-3'
miR-520f Ch-IP 1 sense:	5'-GTCTCGTTCTGTCACCCAGG-3'
miR-520f Ch-IP 1 antisense:	5'-GCATTTATTGGGGCCGGGCGC-3'
miR-520f Ch-IP 2 sense:	5'-ACCTGGTCAAGGAAGATTCCC 3'
miR-520f Ch-IP 2 antisense:	5'-CAGGGACCTTGTCTTGGAATAC-3'

Table S4.The relationship between NEK9 and clinicopathological characteristics in GC

Clinicopathological variables		Total (n=363)	NEK9 expression		*P value*
			Weak expression(-~+) (n=141)	Strong Positive(++~+++) (n=222)	
Gender	Male	225	85	140	0.595
	Female	138	56	82	
Age(y)	≤60	178	75	103	0.207
	>60	185	66	119	
Classification	I+II	146	70	76	0.004
	III+IV	217	71	146	
T stage	T1+T2	122	52	70	0.293
	T3+T4	241	89	152	
N stage	N0+N1	240	106	134	0.004
	N2+N3	123	35	88	
M stage	M0	294	124	170	0.007
	M1	69	17	52	

Table S5. Potential serine phosphorylation sites in ARHGEF2

Name	Sequences
S645	SESLESPRGER
S648	SESLESPR
S691	EPALPLEPDSGGNTSPGVTANGEAR
S932	QELGSPEER
S947	LQDSSDPDTGSEEEGSSRLSPPHSPR
S952	LQDSSDPDTGSEEEGSSRLSPPHSPR
S953	LQDSSDPDTGSEEEGSSRLSPPHSPR
S956	LQDSSDPDTGSEEEGSSRLSPPHSPR
S960	LQDSSDPDTGSEEEGSSRLSPPHSPR

Table S6. Potential STAT3 binding site on miR-520f promoter

Score	Relative score	Start	End	Strand	Predicted site sequence
6.251	0.873282809099598	197	207	-1	CTGCTAAAAAT
5.282	0.861543806120268	294	304	-1	ATGCCTGTAAT
5.282	0.861543806120268	683	693	-1	ATGCCTGTAAT
6.592	0.877413872067556	1517	1527	-1	CTTCAAAGAAT
9.358	0.910922728869234	1569	1579	1	ATTCCAGAAAA
10.162	0.920662830412455	1735	1745	-1	CTACTTGGAAA
5.282	0.861543806120268	1885	1895	-1	CTGCCTGTAAT
6.462	0.875838980026986	1964	1974	-1	TTTGTGGGAAT
5.548	0.864766277526359	2101	2111	-1	TGTCTTGGAAT

Table S7. Primers for miR-520f promoter construct

Name	Sequences
miR-520f-1（-2000~+500bp) sense KpnI:	5'CGGGGTACCTGCGGTGGCGCTTCCAACCTAGACCTCTTAG- 3'
miR-520f-2（-1696~+500bp) sense KpnI:	5'CGGGGTACCGAGCCACCCTGCTCGGACTGCAGGG-3'
miR-520f-3（-1307~+500bp) sense KpnI:	5'CGGGGTACCGAGCCACCGCACCCGGTGAGGAGTTATTT-3'
miR-520f-4（-421~+500bp) sense KpnI:	5'CGGGGTACCCATGCAAACAGGGCCAATAAATGCATCTT-3'
miR-520f-5（-105~+500bp) sense KpnI:	5'CGGGGTACCGAGCCACTGCGCCCGGCCCCAATAAATGC-3'
miR-520f-6 （+111~+500bp) sense KpnI:	5'CGGGGTACCAGGTCCCTGTTGCCCAGGCTGGAGTGCG -3'
Antisense HindIII:	5'CCCAAGCTTAAGCTAAAATCCACATCTCAGAGTTCATCTC-3'

Table S8. Primers for miR-520f promoter site-directed mutagenesis

**Name**	**Sequences**
Binding site 1 mutation sense：	5'TTCTCCTGCCTCAGCGGGTTCCAGCACTGGGGC TACAGGTGCCCACCACCACGCTAGGCT-3'
Binding site 1 mutation antisense:	5'GTAGCCCCAGTGCTGGAACCCGCTGAGGCAGG AGAATGGCGTGAACCCAGGAGGCTGAGC-3'
Binding site 2 mutation sense：	5'TCCCAAAGTGCTGGGCGGCTCAATCGGAGCCA CTGCGCCCGGCCCCAATAAATGCATCTT-3'
Binding site 2 mutation antisense:	5'GTGGCTCCGATTGAGCCGCCCAGCACTTTGGG AGGCCGAGGCGGGCAGATCACGAGGTCA-3'
